# Gadolinium contrast agents: *Over exposure?*

**DOI:** 10.1186/1532-429X-18-S1-T7

**Published:** 2016-01-27

**Authors:** Robert W Biederman, Ronald B Williams, Mark Doyle, June A Yamrozik, Moneal Shah, Geetha Rayarao, Sirikarn Napan

**Affiliations:** grid.413621.30000000404551168Cardiac MRI, Allegheny General Hospital, Pittsburgh, PA USA

## Background

MRI has dramatically changed the way we diagnose disease. With the introduction of contrast agents specifically for MRI, we were now able to see that which was ‘invisible'. Gadopentetate dimeglumine (Magnevist^®^) was introduced over 20 years ago, since then many more agents with variable features have been introduced. Several newer agents have improved ‘relaxivity' and some designated for organ specify use. Along with the increased administration of these agents, has been the emergence of an associated disease complex, Nephrogenic Systemic Fibrosis (NSF). This is an issue only in severely impaired renal function patients(eGFR<30 mL/min/1.73 m^2^). With increased understanding, the incidence of NSF has plummeted. Some agents such as MultiHance^®^ (gadobenate dimeglumine) have never had a single episode of NSF. Most recently a ‘new' issue with gadolinium-based contrast agents has emerged; intracranial gadolinium deposition appearing presumably late only after multiple contrast MRI's. McDonald and Radbruch, noted gadolinium in pts' brain tissue especially in the globus pallidus, thalamus, dentate nucleus and pons in pts with at least four contrast exams. More troubling, as opposed to NSF, this finding was found in pts with preserved GFR (eGFR≥49 mL/min/ 1.73 m^2^).

**Objective**

To perform a real-world temporal analysis of the frequency in which >4 cumulative doses of gadolinium was administered.

## Methods

**Figure 1:**
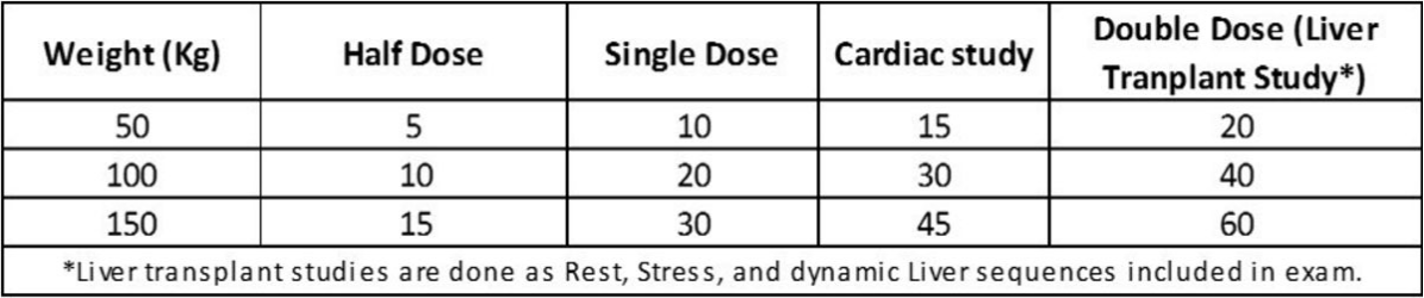
Figure 1

We performed a retrospective examination of our CMR studies to assess the usage of gadolinium (MultiHance) and frequency of exposure to multiple doses normalized by weight. In all cases, we used a weight based dosing formula. Accordingly, we have the following clinical examples for doses showing various administrations: **Doseage=(Kg)(0.1/Kg)(1 ml/0.5 mmol)** or simply:**Doseage=(Kg)(0.2 ml**) for a single dose. Figure 1.

## Results

**Figure 2:**
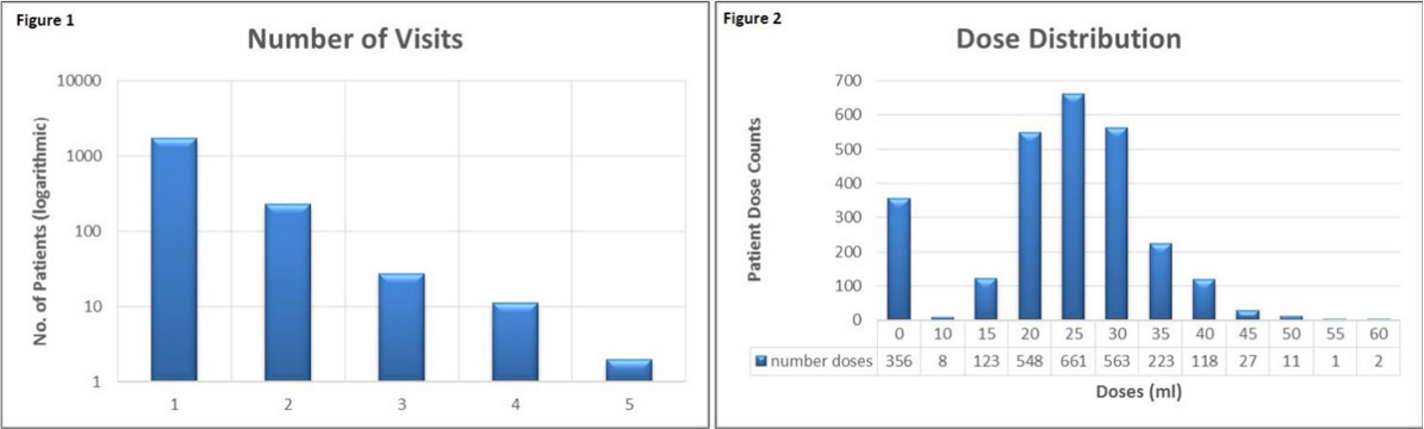
Figure 2

For ~2000 patients given MultiHance (2010-2015), Figure 1 shows the range of gadolinium exposures, revealing a non-Gaussian distribution. In Figure 2, we show a near-Gaussian distribution of dose amounts, peaking at 25 ml.

## Discussion

In our experience, only a small percentage of pts received multiple doses of gadolinium, with ≥4 exposures representing only 0.65% (13/1982) of the sampled population. Assuming similar practice patterns as our high-referral CMR center, the 100^**+**^million doses world-wide pts exposed to gadolinium each year, we suggest that a similar percentage, 0.65%, would receive multiple exposures (>4), and they represent a sufficiently small population that could be followed on a society-wide initiative to better understand brain deposition significance.

## Conclusions

Given the available information in this newly ‘discovered entity' and assuming this has clinical relevance, until a 'better' MRI agent can be found, gadolinium agents still remain the best modality for optimizing clinical diagnoses for MRI sequences. This is especially evident in CMR where infiltrative diseases, MI's and other cardiomyopathies are optimized utilizing these contrast agents.

